# Opposing associations between sedentary time and decision-making competence in young adults revealed by functional connectivity in the dorsal attention network

**DOI:** 10.1038/s41598-020-70679-7

**Published:** 2020-08-19

**Authors:** Dominika M. Pindus, Christopher E. Zwilling, Jennifer S. Jarrett, Tanveer Talukdar, Hillary Schwarb, Evan Anderson, Neal J. Cohen, Aron K. Barbey, Arthur F. Kramer, Charles H. Hillman

**Affiliations:** 1 Department of Kinesiology and Community Health, University of Illinois at Urbana-Champaign, 906 S Goodwin Avenue, Urbana, IL 61801 USA; 2 Beckman Institute for Advanced Science and Technology, University of Illinois at Urbana-Champaign, Urbana, IL USA; 3 Interdisciplinary Health Sciences Institute, University of Illinois at Urbana-Champaign, Urbana, IL USA; 4 Department of Psychology, University of Illinois at Urbana-Champaign, Urbana, IL USA; 5 Department of Psychology, Northeastern University, Boston, MA USA; 6 Department of Physical Therapy, Movement, and Rehabilitation Sciences, Northeastern University, Boston, MA USA

**Keywords:** Neuroscience, Psychology, Human behaviour

## Abstract

How daily physical activity and sedentary time relate to human judgement and functional connectivity (FC) patterns that support them remains underexplored. We investigated the relationships between accelerometer-measured moderate-to-vigorous physical activity (MVPA) and sedentary time to decision-making competence (DMC) in young adults using a comprehensive Adult-Decision Making Competence battery. We applied graph theory measures of global and local efficiency to test the mediating effects of FC in cognitively salient brain networks (fronto-parietal; dorsal attention, DAN; ventral attention; and default mode), assessed from the resting-state fMRI. Sedentary time was related to lower susceptibility to a framing bias. However, once global and local efficiency of the DAN were considered we observed (1) *higher susceptibility* to framing with *more sedentary time,* mediated through lower local and global efficiency in the DAN, and (2) *lower susceptibility* to framing with *more sedentary tim*e. MVPA was not related to DMC or graph theory measures. These results suggest that remaining sedentary may reduce neurofunctional readiness for top-down control and decrease engagement of deliberate thought, required to ignore irrelevant aspects of a problem. The positive effect suggests that the relationship between sedentary time and DMC may be moderated by unmeasured factors such as the type of sedentary behavior.

## Introduction

Physical inactivity and excessive sedentary time are associated with an increased risk of cognitive decline due to increased cardiometabolic risk^1^. However, in working age adults the relevance of physically inactive and sedentary lifestyles to higher order cognition such as reasoning and decision-making remains poorly understood. Aerobic exercise interventions are effective at improving cognitive control (a set of cognitive processes that schedule, control and maintain goal directed behavior) across the lifespan^2–4^. Recent evidence extends these positive effects to decision-making competence (DMC)^5^. DMC involves evaluating the probabilities (i.e. belief assessments) and desirability (i.e. value assessment) of different choices, and integrating this information while recognizing the limits of one’s own knowledge^6^. Each aspect is evaluated through specific components (tests) reflecting individual’s capacity to overcome common cognitive biases in decision-making and thus higher DMC. Using a randomized controlled trial, we recently evidenced the efficacy of a high intensity fitness training intervention in improving several aspects of DMC that rely on cognitive control (e.g., meta-cognitive and integrational aspects), in young healthy adults^5^.

However, the generalizability of the findings from aerobic exercise interventions remains limited because almost a half of young adults in the USA are physically inactive (i.e., defined as accumulating < 150 min of moderate or < 75 min of vigorous physical activity per week or less than the equivalent combination of the two intensities)^7,8^. Furthermore, individual differences in sedentary time have not been controlled for in these studies, while poorer general cognitive functioning was observed with higher sedentary time among older adults, regardless of moderate-to-vigorous physical activity (MVPA)^9^. Sedentary behavior is a distinct from physical inactivity behavior^10^. It is defined as the time spent in a sitting or reclined position with energy expenditure not exceeding 1.5 metabolic equivalents^10^ (one metabolic equivalent is the rate of energy expenditure while sitting at rest expressed as oxygen uptake of 3.5 mL kg^−1^ min^−1^ in adults^11^). How daily MVPA (comprising incidental and structured physical activities) and sedentary time contribute to complex cognitive abilities such as DMC in young, working age adults remains to be determined. The associations between sedentary time, DMC and functional connectivity are expected to be independent of engagement in MVPA because an individual can be concurrently physically active (i.e., by adhering to physical activity recommendations^12^) and highly sedentary (e.g., by engaging in ≥ 8 h of sedentary time per day^13^). In confirmation, prior research has shown adverse associations between objectively measured sedentary time^14^, self-reported TV viewing, passive commuting^9^ and general cognitive ability after controlling for MVPA.

Seminal meta-analytical findings suggest more pronounced effects of physical activity on tasks that engage cognitive control^15^, a robust correlate of higher DMC^16,17^. Indeed, adults with higher levels of daily MVPA demonstrate enhanced cognitive control^18,19^. The benefits of daily MVPA could thus extend to DMC, specifically to these aspects that to a greater extent rely on cognitive control such as resistance to framing (the ability to overcome the effects of positive or negative valence frames), following complex decision rules when weighing options and ability to recognize limits of one’s knowledge^20^. Greater engagement in sedentary behaviors (e.g., more time spent watching TV or engaging in sedentary commuting) has been related to poorer fluid intelligence^14^ and cognitive control^9^ in epidemiological and longitudinal studies. Higher sedentary time may therefore adversely affect DMC by downregulating cognitive control in favor of automatic information processing and increasing susceptibility to well-known cognitive biases (such as a framing bias) in decision-making^21^.

Research in network neuroscience reveals that complex cognitive abilities (such as cognitive control^22^, intelligence^22,23^ and DMC^24^) rely on co-activation of distributed brain regions (i.e., fronto-parietal regions) that support flexible modulation of cognitive control and goal-directed behavior^25,26^. These co-activation patterns can be measured at rest using resting state functional magnetic resonance imaging (fMRI). Resting state fMRI is used to identify correlations between BOLD signal fluctuations in brain regions supporting higher order cognition, thought to represent individual differences in the ability to engage cognitive control^27^. Increased cognitive control^25,28–30^, higher intelligence^23^ and DMC^24^ have been associated with increased resting state functional connectivity (FC) in four intrinsic connectivity brain networks: fronto-parietal network (FPN; comprising dorsolateral prefrontal and dorsal frontal cortices, inferior parietal lobule, intraparietal sulcus, precuneus and middle cingulate cortex) which initiates and adjusts control; dorsal attention network (DAN; comprising frontal eye field, and parts of intraparietal cortex) which directs top-down control of attention; ventral attention network (VAN; including temporo-parietal junction and ventral frontal cortex) which re-orients attention to relevant and salient aspects of the environment^29^; and default mode network (DMN; comprising ventral medial prefrontal and posterior cingulate cortices^31^) with better performance observed on working memory^32^ and inhibitory control tasks^33^ with stronger resting state FC in DMN.

Greater plasticity of resting state FC patterns in these networks^34,35^ may render them amenable to physical activity interventions^36^, individual differences in aerobic fitness^37^ and daily physical activity^38^ in older adults. In younger adults, greater resting state FC in brain regions sensitive to individual differences in aerobic fitness correlated with higher resting state FC in FPN, DAN, VAN and DMN^37^. In turn, stronger resting state FC in FPN and VAN was associated with better DMC^24^. Thus, increased resting state FC in cognitively salient brain networks may mediate the association between a physically active lifestyle and higher DMC. However, aerobic fitness is only a proxy for a physically active lifestyle and shows only moderate correlations with objectively measured MVPA^39^ (in part due to its large genetic component^40^). How a sedentary lifestyle may affect resting state FC patterns in cognitively salient networks among young adults remains largely underexplored.

Beyond the resting state FC patterns, graph theory measures assess the efficiency of information processing within and across intrinsic connectivity brain networks. In graph theory, the brain is viewed as a complex network comprising edges and vertices. Biologically plausible metrics are used to estimate the efficiency of information processing within specific and functionally distinct brain regions (measures of network segregation), and parallel information processing using measures of system integration. Local efficiency (a measure of system segregation), a proxy for information exchange within specialized brain modules (i.e., brain regions), measures how densely connected the nodes are within a module^41,42^. Global efficiency (a measure of network integration) represents the shortest functional connections between distant brain regions and serves as a proxy for parallel information processing in the brain^41–43^.

Local efficiency in cognitively salient networks (FPN, DAN, VAN and DMN) is a sensitive marker of functional integrity^44^ and cognitive reserve^45^. More aerobically fit^46^, and more physically active older adults^47^ show greater local efficiency in these networks. Aerobic fitness also benefits global efficiency in older adults^46,48^. Whether the benefits of aerobic fitness (a physical state) for local and global efficiency in cognitively salient brain networks extend to physical activity *behavior* remains largely underexplored. One study reported no association between physical activity and global efficiency across cognitively salient brain networks in older adults^47^. However, the use of physical activity questionnaires may have occluded the underlying associations due to inaccuracies inherent in self-reported physical activity^49^. Global and local efficiency in cognitively salient brain networks are good candidates for mediators of the relationship between daily MVPA, daily sedentary time and DMC due to strong associations of global efficiency to general intelligence^23^, and positive relationship between local efficiency, cognitive control and memory^45^.

This study had two aims: first, to assess the relationships between objectively measured MVPA, sedentary time and DMC in young adults; and second, to test the mediating effects of FC in the FPN, DAN, VAN and DMN on these relationships. In relation to aim 1, we hypothesized that young adults who engaged in more daily MVPA would show better DMC. We expected that the relationships between MVPA and DMC would be independent of sedentary time because (1) aerobic exercise interventions yield no clinically meaningful changes in daily sedentary time^50,51^, thus their positive effects on cognitive functions could not be explained by concurrent decrements in sedentary time; and (2) because high volume of sedentary time has been related to increased cardiometabolic^52,53^ and cardiovascular risk even after controlling for MVPA^54,55^.

Based on the emergent evidence of poorer cognitive control with greater engagement in sedentary behaviors^56^, we hypothesized that more sedentary young adults would show poorer DMC. We predicted that these associations would persist after controlling for MVPA based on the findings that sedentary time-related increases in cardiovascular and metabolic health risks are largely independent of MVPA^13^. In relation to aim 2, we predicted that more daily MVPA would be related to greater global and local efficiency in cognitively salient brain networks (FPN, DAN, VAN and DMN), while more sedentary time would be related to lower global and local efficiency in these networks. Next, we predicted that the relationships between MVPA, sedentary time and performance on DMC tests would be mediated through the associations of these physical behaviors with FC in cognitively salient brain networks. Lastly, we predicted that these mediating effects would hold once the differences in sedentary time and MVPA were statistically controlled.

## Results

Sample characteristics are presented in Table 1. MVPA was negatively and moderately related to sedentary time (*ρ* = − 0.35, *p* < 0.001), which justified mutual adjustments for sedentary time and MVPA in the regression models. MVPA was positively but weakly related to fat free peak oxygen uptake (FFVO_2_peak; *ρ* = 0.23, *p* = 0.001), while sedentary time was not related to FFVO_2_peak (*p* = 0.39).

**Table 1:** Sample characteristics (N = 201).

Demographics	M (SD)^a^
Age (years)	24.2 (5.3)
Sex (n, (%) females)	104 (52)
**Education (n, (%))**	
High school^b^	102 (51)
Bachelor’s degree or higher^c^	99 (49)
**Intelligence quotient**	
Shipley vocabulary	112.2 (8.8)
Figure series (n correct; max. 30)	2.4 (1.2)
BMI (kg m^−2^)	24.2 (3.9)
Overweight or obese (n, (%))	69 (34)
FFVO_2_peak (mL kg^−1^ min^−1^)	57.1 (7.3)
**VO**_**2**_**peak percentile**	
Low fit (< 35%-tile^d^; n(%))	70 (35)
**Accelerometry**	
Valid days of accelerometer wear [Med (IQR)]	6.0 (2.0)
Accelerometer wear time (min/day)	865.9 (119.4)
Sedentary time (min/day)	603.6 (119.2)
MVPA (min/day)	41.3 (24.8)
Meeting physical activity guidelines (n, %)	159 (79)
**Local efficiency (range 0–1)**	
FPN	0.35 (0.03)
DAN	0.35 (0.03)
VAN	0.38 (0.04)
DMN	0.32 (0.02)
**Global efficiency (range 0–1)**	
FPN	0.28 (0.04)
DAN	0.30 (0.04)
VAN	0.32 (0.04)
DMN	0.26 (0.03)
**A-DMC**	
A-DMC index (z score)	0.08 (0.52)
Over/underconfidence (range 0–1)	0.92 (0.06)
Applying decision rules (range 0–1)	0.84 (0.15)
Consistency in risk perception (range 0–1)	0.75 (0.11)
Recognizing social norms (range −1 to 1)	0.48 (0.21)
Resistance to framing (range 0–5)	4.20 (0.43)
Resistance to sunk cost (range 0–6)	4.36 (0.69)

*BMI* body mass index; overweight was defined based on BMI ≥ 25 and < 30, obesity was defined as BMI ≥ 30^133^; *FFVO*_*2*_*peak* peak oxygen uptake adjusted for fat free mass, *MVPA* moderate-to-vigorous physical activity, *DAN* dorsal attention network, *VAN* ventral attention network, *FPN* frontoparietal network, *DMN* default mode network, *A-DMC* Adult-Decision Making Competence Battery.

^a^Number and percent are included for categorical variables; median values are presented for the number of valid days; ^b^includes 1 participant with vocational school; ^c^includes 11 participants with an associate’s degree; ^d^^11^.

### Moderate-to-vigorous physical activity, sedentary time and decision—making competence

**Figure 1:**
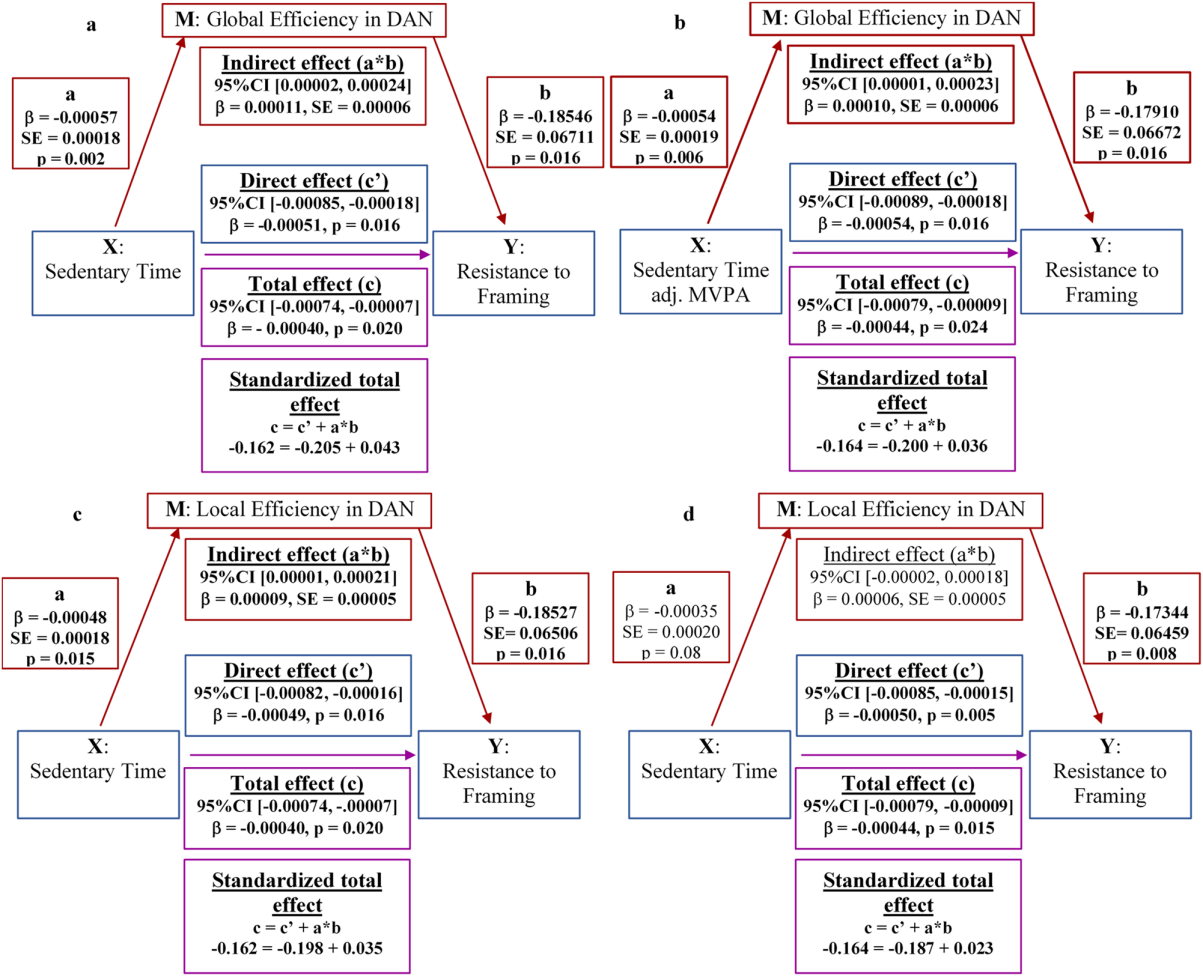
Direct and mediated effects of sedentary time on the ability to resist framing bias: (**a**) sedentary time *unadjusted* for moderate-to-vigorous physical activity (MVPA) and a mediating effect of global efficiency of the dorsal attention network (DAN); (**b**) sedentary time *adjusted* for MVPA and a mediating effect of global efficiency of the DAN; (**c**) sedentary time *unadjusted* for MVPA and local efficiency of the DAN; (**d**) sedentary time *adjusted* for MVPA and a mediating effect of local efficiency of the DAN; standardized total effects illustrate the reduction of the total positive effect of sedentary time (path c) on the ability to resist framing due to a negative indirect effect (paths a*b) carried by the decrease in the global and local efficiency in the DAN with increasing sedentary time (path a). The directionality of the effects in relation to resistance to framing is reversed: negative betas denote positive associations (paths c, c′, b) because values for resistance to framing are reflected and transformed (using Tukey power transformations). Blue: a direct effect of a predictor (X) on the outcome (Y), adjusting for the mediator (M); red: an indirect effect of X on Y via M; magenta: the total effect of X on Y.

All subsequent *p *values reported in the text have been adjusted for False Discovery Rate, (FDR)^57^; uncorrected results are presented in Supplemental Table S1. MVPA was not related to performance on any of the DMC tests measured with adult decision-making competence (A-DMC) battery (*p*s ≥ 0.08). In contrast, sedentary time was positively related to resistance to framing (i.e., indicating a positive relationship between sedentary time and cognitive performance) in unadjusted as well as adjusted for MVPA models (*p*s = 0.02; path c, Fig. 1a–c), but not to other A-DMC tests (*p*s > 0.10). Note that negative betas in Fig. 1 indicate *a positive relationship* between sedentary time and resistance to framing because scores on resistance to framing were negatively skewed and required transformations to adhere to normality (i.e., scores were first reflected by subtracting each score from the highest score plus 1 and then transformed). Consequently, *higher transformed scores* indicate *lower resistance to framing* and thus poorer cognitive performance. To avoid confusion, we provide a conceptual interpretation of statistical results in the text such that *negative betas* are interpreted as *a positive relationship* between sedentary time and resistance to framing (i.e., indicating better cognitive performance with higher sedentary time), and *positive betas* are interpreted as *a negative relationship* (i.e., indicating poorer cognitive performance with higher sedentary time). We next assessed mediating effects models. First, the relationships between MVPA, sedentary time and hypothesized mediators (local and global efficiency in FPN, DAN, VAN and DMN) were explored.

### Step 1: Sedentary time, moderate-to-vigorous physical activity and graph theory measures

Tables 2 and 3 summarize the results of the regression models predicting global and local efficiency of brain networks from MVPA and sedentary time (***Supplemental Tables S2, S3 present models with uncorrected for FDR values). MVPA was not related to either global (*p*s ≥ 0.17) or local (*p*s ≥ 0.10) efficiency across all brain networks irrespective of adjustment for sedentary time. In contrast, more time spent sedentary (unadjusted for MVPA) was related to lower global efficiency [*F*(8, 192) = 2.65, *p* = 0.01] and lower local efficiency [*F*(8, 192) = 2.08, *p* = 0.04] in the DAN. Adjusting for MVPA did not offset the decrease in global efficiency of the DAN associated with more time spent sedentary [*F*(8, 192) = 2.32, *p* = 0.02] but it did offset the decrease in local efficiency (*p* = 0.16). These results suggest that sedentary time may be a more sensitive behavioral marker of global and local efficiency in the DAN compared to MVPA in young adults.

**Table 2:** Global efficiency of intrinsic connectivity networks regressed on daily moderate-to-vigorous physical activity and sedentary time.

	DAN	VAN	FPN	DMN
**Sedentary time**				
∆R^2^	**0.0488**	0.0207	0.0095	0.0035
β	**− 0.0006**	− 0.0003	− 0.0002	− 0.0002
(SE)	**0.0002**	0.0002	0.0001	0.0002
b	**− 0.2266**	− 0.1476	− 0.0999	− 0.0605
**Sedentary time adjusted for MVPA**				
∆R^2^	**0.0375**	0.0287	0.0107	0.0073
β	**− 0.0005**	− 0.0004	− 0.0002	− 0.0002
(SE)	**0.0002**	0.0002	0.0002	0.0002
b	**− 0.1963**	− 0.1720	− 0.1047	− 0.0864
**MVPA**				
∆R^2^	0.0114	0.0017	0.0000	0.0032
β	0.0008	− 0.0003	0.0000	− 0.0004
(SE)	0.0005	0.0005	0.0004	0.0005
b	0.1108	− 0.0424	0.0015	− 0.0587
**MVPA adjusted for sedentary time**				
∆R^2^	0.0007	0.0103	0.0014	0.0071
β	0.0002	− 0.0007	− 0.0002	− 0.0006
(SE)	0.0005	0.0005	0.0004	0.0005
b	0.0260	− 0.1038	− 0.0384	− 0.0861

Global efficiency in intrinsic functional brain networks was transformed using Tukey power transformations. All physical behavior variables were adjusted for accelerometer wear time.

Bolded values indicate significant associations at *p* < 0.05 after FDR correction. Model: Global efficiency of a brain network = age + sex + education + BMI + IQ + FFVO_2_peak + MVPA/Sedentary time.

*MVPA* moderate-to-vigorous physical activity, *DAN* dorsal attention network, *VAN* ventral attention network, *FPN* frontoparietal network, *DMN* default mode network.

**Table 3:** Local efficiency of intrinsic connectivity networks regressed on daily moderate-to-vigorous physical activity and sedentary time.

	DAN	VAN	FPN	DMN
**Sedentary time**				
∆R^2^	**0.0329**	0.0004	0.0002	0.0015
β	**− 0.0001**	− 0.0000	− 0.0000	0.0000
(SE)	**0.0000**	0.0000	0.0001	0.0000
b	**− 0.1861**	− 0.0194	− 0.0127	0.0392
**Sedentary time adjusted for MVPA**				
∆R^2^	0.0158	0.0029	0.0003	0.0005
β	− 0.0001	− 0.0000	− 0.0000	0.0000
(SE)	0.0000	0.0000	0.0002	0.0000
b	− 0.1276	− 0.0546	− 0.0166	0.0226
**MVPA**				
∆R^2^	0.0296†	0.0074	0.0001	0.0022
β	0.0003	− 0.0001	− 0.0000	− 0.0001
(SE)	0.0001	0.0001	0.0004	0.0001
b	0.1783	− 0.0890	− 0.0084	− 0.0489
**MVPA adjusted for sedentary time**				
∆R^2^	0.0123	0.0098	0.0002	0.0012
β	0.0002	− 0.0002	− 0.0001	− 0.0000
(SE)	0.0001	0.0001	0.0004	0.0001
b	0.1134	− 0.1016	− 0.0140	− 0.0358

Local efficiency in frontoparietal networks was transformed using Tukey power transformations. Data for all other intrinsic connectivity brain networks was untransformed. All physical behavior variables were adjusted for accelerometer wear time.

Bolded values indicate significant associations at *p* < 0.05 after FDR correction. Model: Global efficiency of a brain network = age + sex + education + BMI + IQ + FFVO_2_peak + MVPA/Sedentary time.

*MVPA* moderate-to-vigorous physical activity, *DAN* dorsal attention network, *VAN* ventral attention network, *FPN* frontoparietal network, *DMN* default mode network.

### Step 2: Graph theory measures and decision-making competence

To satisfy the second condition of the mediation^58^, we tested if global and local efficiency in the DAN (i.e., the hypothesized mediators; path b, Fig. 1a–c) were related to performance on A-DMC battery (outcomes) when adjusting for sedentary time (a hypothesized predictor). Global efficiency of the DAN was related to higher resistance to framing [△*R*^2^ = 0.03, β = − 0.19, *SE* = 0.07, *b* = − 0.19, *p* = 0.02, *F*(9, 191) = 5.17, *p* < 0.001], as did local efficiency [△*R*^2^ = 0.03, β = − 0.19, *SE* = 0.07, *b* = − 0.19, *p* = 0.02, *F*(9, 191) = 5.23, *p* < 0.001]. Note that negative betas indicate a positive relationship. Neither global nor local efficiency in the DAN were related to the remaining A-DMC tests (*p*s ≥ 0.22). Next, we formally tested the mediating effects of global and local efficiency in the DAN on the relationship between sedentary time and resistance to framing.

### Steps 3: Sedentary time and decision-making competence—mediating effects

**Figure 2:**
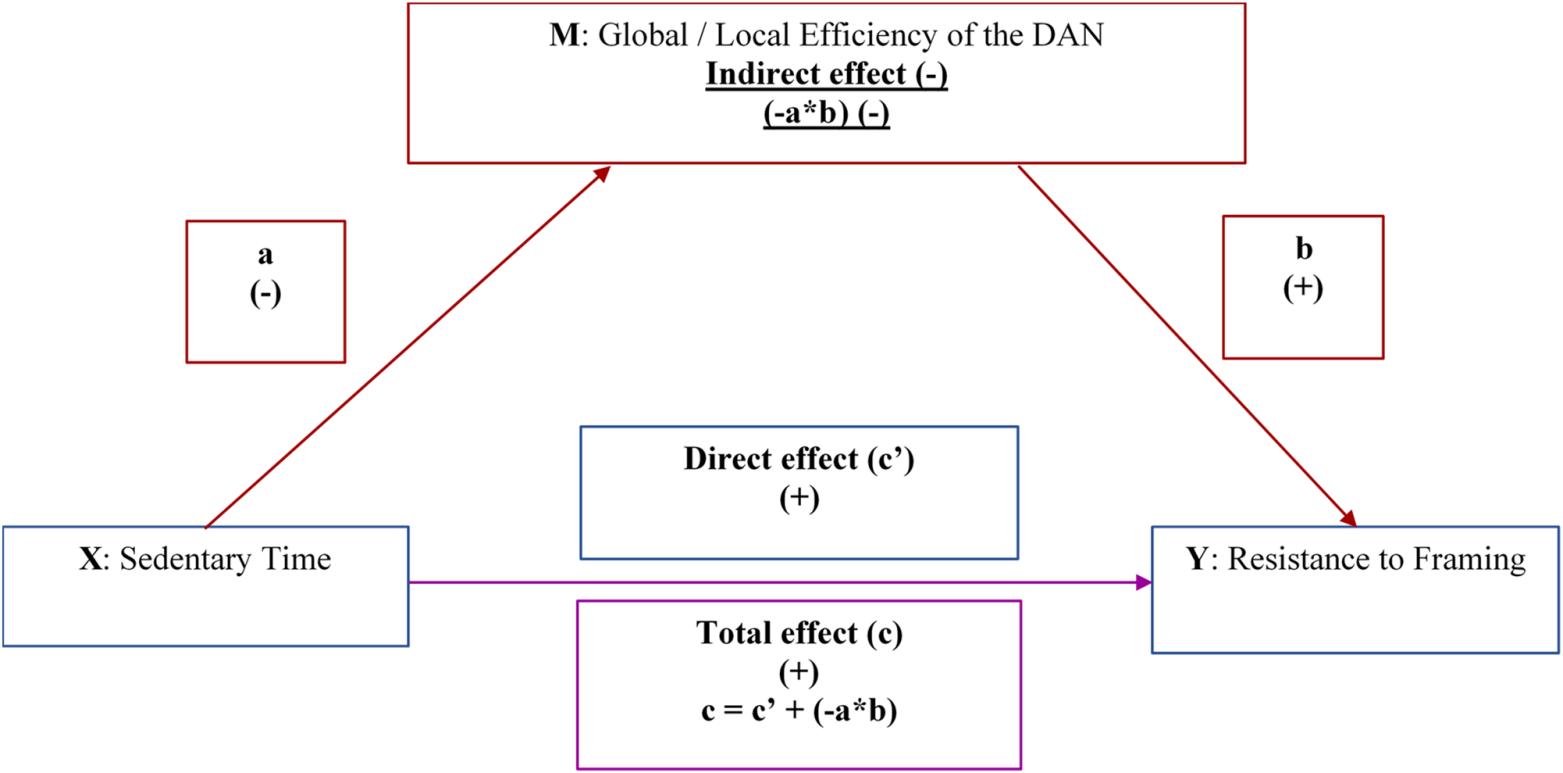
A graphical summary of the directionality of mediating and direct effects of sedentary time on resistance to framing through global and local efficiency of the dorsal attention network (DAN). Blue: a direct effect of a predictor (X) on the outcome (Y), adjusting for the mediator (M); red: an indirect effect of X on Y via M; magenta: the total effect of X on Y.

In contrast, mediating effects models revealed two opposing statistical effects of sedentary time on resistance to framing: (1) *an indirect effect,* where sedentary time was *negatively* related to resistance to framing through its effect on global (a*b: β = 0.00011, 95% bootstrap *CI* [0.00002, 0.00024]) and local efficiency of the DAN (a*b: β = 0.00009, 95% bootstrap *CI* [0.00001, 0.00021]; Fig. 1a,c), and (2) *a positive direct effect* of sedentary time on resistance to framing, controlling for global (c′: β = − 0.00051, 95% *CI* [− 0.00085, − 0.00018], Fig. 1a) and local efficiency of the DAN (c′: β = − 0.00049, 95% *CI* [− 0.00082, − 0.00016], Fig. 1c). We remind the reader that positive betas in the indirect effects indicate a negative relationship between sedentary time and resistance to framing through global (and local) efficiency of the DAN, while negative betas in the direct effects of sedentary time on resistance to framing indicate a negative relationship. Controlling for MVPA suppressed the negative indirect effect of the local (95%* CI* [-0.00002, 0.00018]; Fig. 1d) but not global efficiency in the DAN (95% *CI* [0.00001, 0.00023]; Fig. 1b). The positive direct effect of sedentary time on resistance to framing after controlling for MVPA also remained statistically significant (FDR-corrected *ps* = 0.02, Fig. 1b).

Taken together, these results suggest that sedentary time acted on resistance to framing through two opposing pathways: one through *decreased global and local efficiency* in the DAN, leading to a *lower* resistance to framing, and a second one that promoted *enhanced* resistance to framing, potentially due to other mediators which were unmeasured in this study. Greater engagement in MVPA attenuated the negative mediating effect of decreased local but not global efficiency in the DAN on the relationship between sedentary time and resistance to framing. The negative indirect effects carried via global and local efficiency in the DAN attenuated but did not suppress the total positive effect of sedentary time on resistance to framing.

## Discussion

Little is known about how daily physical activity and sedentary time relate to applied aspects of higher order cognition such as DMC, and what mechanisms may underpin this relationship. Here, we assessed the relationship between objectively measured daily MVPA and sedentary time to DMC in young healthy adults using the most comprehensive battery of tests to capture each of its core domains^6^. Contrary to our hypothesis, we found that more time spent sedentary was related to higher ability to resist a common cognitive bias in decision-making, a framing bias. However, when FC in cognitively salient brain networks was considered, more sedentary young adults showed lower efficiency of information processing selectively within the DAN (i.e., lower local efficiency) and across the DAN and the remaining brain networks (i.e., lower global efficiency), suggesting lower readiness for top-down control^29,30^. We next probed a hypothesized mechanism underlying the relationship between sedentary time and DMC by formally testing the mediating effect of global and local efficiency in the DAN on this relationship. Mediation analyses revealed two directionally opposing associations. Firstly, when lower global and local efficiency in the DAN (associated with more sedentary time) were considered, more sedentary young adults were less able to resist framing (because of higher resistance to framing with higher global and local efficiency in the DAN). Engaging in more daily MVPA was protective against poorer ability to resist framing due to lower local efficiency but not due to global efficiency in the DAN. However, the mediating effects of global and local efficiency in the DAN did not fully explain the relationship between sedentary time and resistance to framing. Specifically, more sedentary young adults were better able to resist framing even after considering the decrease in resistance to framing with lower FC in the DAN. This relationship was independent of individual differences in daily MVPA. Lastly, in contrast to sedentary time, daily MVPA was not related to either DMC or global and local efficiency in any of the cognitively salient brain networks.

Contrary to our hypothesis we found that sedentary time was positively related to a specific aspect of DMC, the ability to resist framing bias, before (i.e., the total effect, path c Fig. 2) and after controlling for global and local efficiency in the DAN (i.e., a direct effect, path c′ in Fig. 2). Although this result may seem counterintuitive at first, epidemiological evidence suggests that the type of sedentary behaviors may moderate the relationship between sedentary time and higher order cognitive functions^14,59–61^. Specifically, computer use has been related to better cognitive control (task switching and working memory)^14,59–61^, while TV viewing has been related to poorer cognitive control and fluid intelligence^14,59^. Resistance to framing requires deliberate thought to attend to normatively relevant aspects of the problem and ignore irrelevant ones^6^, and therefore relies on cognitive control (working memory)^20^. While accelerometers do not measure the type of sedentary behaviors, epidemiological data suggest that leisure time computer use is three times higher among adults with post-secondary education^62^. Almost half of our participants (49%) had an associate’s or a bachelor’s degree, thus we speculate that they likely engaged in more computer use than TV viewing, which may have driven our positive findings^14,56^. As to the type of computer use, internet browsing accounts for the greatest propotion of computer use in young adults^63^ and can help optimize neural activation in dorsolateral prefrontal cortex^64^. In turn, dorsolateral prefrontal cortext supports the integration of cost and benefit analyses^65^, which is integral to resistance to framing^66^.

To explore a potential mechanism of the relationship between sedentary time and DMC, we tested the mediating effects of global and local efficiency in cognitively salient brain networks (FPN, DAN, VAN and DMN), which have been related to DMC^24^, intelligence^22,23^ and cognitive control^67,68^. Contrary to the total effect of sedentary time on resistance to framing (i.e., before accounting for FC in the DAN), our mediation analyses revealed a negative association between sedentary time and resistance to framing carried through lower global and local efficiency, specifically in the DAN. This result aligns with previous research linking individual differences in resistance to framing to cortical surface area of the dorsal anterior cingulate cortex^17^, a region implicated in cognitive control^69^. The negative association between sedentary time and resistance to framing carried through decreased global and local efficiency in the DAN may reflect experience-dependent readiness of the DAN to engage top-down control. Global efficiency of the DAN is maximized during high network coupling (i.e., the periods when temporal correlations in FC among the network nodes are strong)^70^. High network coupling within the DAN during rest is thought to represent experience-dependent patterns of connectivity^71,72^, largely consistent with FC patterns during cognitive effort^71,73^. For example, lower local efficiency at rest within several nodes of the DAN has been related to poorer performance on a task of visual attention^73^. Thus, lower global and local efficiency of the DAN may render individuals more susceptible to a framing bias by potentially allowing for top-down processes to be initiated too late to stop or override automatic (and consistent with the frame) response.

The significance of this relatively small decrease in resistance to framing with decreased global efficiency of the DAN can be understood in view of the physiologically meaningful changes in sedentary time and age-related decline in resistance to framing^74^. Sedentary time was expressed in minutes per day, while a meaningful change in sedentary time requires displacing higher volumes of sedentary time. For example, substituting two hours of sedentary time with light physical activity attenuated metabolic risk among young adults by 0.09 SD^75^. Accordingly, we found that a 2-h increase in sedentary time (mediated through lower global efficiency in the DAN: a*b = 0.04 or 0.08 SD, a positive beta value indicates a negative relationship) would correspond to twice the rate of the decrease in resistance framing over five years due to aging (b =− 0.02)^74^. The rate of the decrease in global efficiency in the DAN associated with two more hours of daily sedentary time (0.017 units or 4% relative to a maximum in unadjusted and adjusted for MVPA models) in our study is equivalent to age-related decrease in global efficiency of the brain network from young adulthood (19–30 years) to middle age (51–60 years)^76^. Taken together, our results suggest that decreasing sedentary time by at least two hours (i.e., the time spent daily watching TV by 84% of Americans^62^), may protect from suboptimal global (and local) efficiency in the DAN and susceptibility to the framing bias. While small on an individual level, this effect is likely to result in a large number of affected individuals in the population^77^ due to high prevalence of sedentary lifestyles among adults in the USA who spend on average 8 h/day sedentary^78^. Given the ubiquitous role of resistance to framing in daily decision making including career choices^79^, medical decisions^80^ and financial planning^81,82^ and dependence of general cognitive ability on global efficiency of the brain network^23,45^, future research should test the effects of reducing sedentary time on functional connectivity of cognitively salient brain networks and susceptibility of decision-makers to common cognitive biases across adult lifespan.

One potential mechanism that may help explain the negative mediated effect of sedentary time on resistance to framing through global efficiency in the DAN is decreased insulin sensitivity due to high volumes of sitting^83,84^. Greater volume of sedentary time can increase the risk of insulin resistance^85^, while engaging in prolonged sitting (e.g., >  20 min) acutely increases post-prandial insulin response^86^, which may translate to an acute decrease in insulin sensitivity^87^. Peripheral insulin sensitivity can affect the transfer of peripheral insulin to the brain^88,89^, and thus contribute to suboptimal global and local efficiency in the brain networks that rely on frontal lobe (which is sensitive to variation in cerebral insulin^90,91^) including the DAN. Adults with low insulin sensitivity have shown decreased task-related activity in hub regions of the DAN (including parietal and temporal cortices)^92,93^, and difficulty shifting connectivity patterns from reward to cognitive control based networks^94^, while attenuated cerebral glucose metabolism in these regions has been related to poor cognitive control^92^.

In contrast to sedentary time, daily MVPA was not related to FC of cognitively salient brain networks or to DMC. Too small variance in daily MVPA in our sample of healthy and physically active young adults may have contributed to these null findings. Few participants in our sample were physically inactive as 79% met the aerobic component of current physical activity recommendations^12^ and 40% were classified as “highly active” (≥ 300 min of moderate and vigorous per week). Prior research found similar null association between accelerometer-measured MVPA and FC in the DAN in a group of relatively highly active older adults (daily MVPA: *Med* = 33 min/day)^95^. Consistent with curvilinear dose–response relationships of MVPA with several health outcomes related to cognitive control (e.g. mental health^96^, glycated hemoglobin^97^), stronger relationship between physical activity and FC in brain networks and DMC could be expected at a lower end of MVPA continuum, which was not captured in our study. Alternatively, neurocognitive gains may require structured aerobic exercise or high intensity fitness training, consistent with positive effects of aerobic exercise interventions on DMN and frontal-executive network^36^, and high intensity fitness training on DMC^5^.

### Limitations

Our results need to be considered in view of study limitations. We did not capture the modality of sedentary behaviors that young adults engaged in. Thus, we were unable to identify specific sedentary behaviors which may have driven the positive relationship between sedentary time and resistance to framing. Therefore, future studies should include sensitive measures of the modality of sedentary behavior that could be temporally paired with accelerometry data, such as a 24-h recall^98^. Next, while hip-worn accelerometers provide objective measurement of physical movement, their accuracy to assess posture is limited^99,100^. Thus, some misclassification of sedentary time for light physical activity (LPA, and vice versa) was to be expected^101^. However, it is unlikely that misclassification of sedentary time for LPA by accelerometers has affected our conclusions because LPA was negatively related to global efficiency of the DAN and resistance to framing in post-hoc exploratory analyses (***Supplemental Tables S4, S5; results uncorrected for FDR). Next, our sample included young, well-educated and, on average, physically active adults, which may limit generalizability of our findings. To increase generalizability, we controlled for age, sex, education, IQ and FFVO_2_peak (estimated using a gold standard maximal exercise test) in all our analyses because these factors have been related to DMC in previous research. We also mutually controlled for MVPA and sedentary time in our models. Lastly, the observational design precludes causal inferences, and randomized controlled trials specifically designed to test the effects of decreasing sitting time on FC in cognitively salient intrinsic connectivity brain networks and on higher order cognitive functions are needed to establish causality.

## Conclusions

Investigations linking lifestyle behaviors to cognitive functions aim to identify behavioral targets that help optimize mental functioning of the population. Our study reveals how reducing sedentary time may help decrease susceptibility to a framing bias by optimizing functional connectivity patterns that underpin top-down attentional control. Framing bias prompts a decision maker to accept greater risks to avoid losses across contexts including career seeking and financial planning. As such, we show that sedentary lifestyle may subtly affect a broad range of human behaviors. A net positive association between sedentary time and resistance to framing suggests that young and well-educated adults in our study may have primarily engaged in cognitively enhancing sedentary behaviors which decreased their susceptibility to a framing bias. By revealing the complexity of the relationship between sedentary time and resistance to framing, our results emphasize the need to consider the modality of sedentary behaviors and their neurofunctional correlates in future studies. Thus, our study paves the way for future research into the role of sedentariness in decision-making competence and its neurofunctional signatures.

## Methods

### Population

Baseline data from adults participating in the INSIGHT trial (ClinicalTrials.gov: NCT02780739)^5^ were included in the study. The study adhered to the Code of Ethics of the World Medical Association^102^, and the University of Illinois IRB approved the study protocol; all participants provided informed consent. As described previously^5^, we included adults: (1) aged 18–44 years; (2) fluent in speaking English; (3) with normal or corrected-to-normal vision and hearing; free from: (4) medications affecting central nervous system; (5) psychological, neurological, or endocrine disease; or (6) history of concussion within the past two years; or (7) learning disorders, (8) if a smoker, < 10 cigarettes per day; (9) with a body mass index (BMI) < 35; and (10) no contraindications to exercise as per revised Physical Activity Readiness Questionnaire^103^. After exclusions based on insufficient accelerometer wear (i.e. < 4 days; n = 98)^104^, missing data on fMRI (n = 93), fat free VO_2_peak (FFVO_2_peak; n = 1), missing or low scores (< 85) on Shipley-2 intelligence test^105^ (n = 34), missing education (n = 2), 201 participants (*M*_age_ = 24.2, *SD* = 5.3 years, age range [18.0, 43.9]; 47% out of the original sample N = 429) were included.

### Accelerometry

Physical activity and sedentary time were measured using a triaxial wGT3X-BT accelerometer (ActiGraph LLC., Pensacola, FL, USA), a small device (3.3 × 4.6 × 1.5 cm; 19 g) with a dynamic range of ± 8 g. Sampling frequency was set to 100 Hz. Accelerometers were worn on an elastic belt at the right anterior axillary line for 7 consecutive days during waking hours except for water-based activities. Non-wear time (60 consecutive minutes of 0 counts^106,107^) was excluded from the analyses. Only participants with ≥ 4 days of ≥ 10 h of wear time were included^104,108^. Acceleration signal was converted into vertical axis counts over 60 s epochs and time (min/day) spent sedentary (< 100 CPM^109^) and in MVPA (≥ 2,020 CPM^110^) was calculated using ActiLife software (version 6.13.3; ActiGraph LLC., Pensacola, FL, USA).

### fMRI data acquisition and processing

All images were acquired using Siemens Magnetom 3 T Trio scanner using a 32-channel head coil and processed exactly as previously described^24,37^. High resolution structural images were acquired using multi-echo T1-weighted magnetization prepared gradient echo (0.9 mm isotropic resolution, TR = 1900 ms, TI = 900 ms, TE = 2.32 ms, with GRAPPA and an acceleration factor of 2)^111^. Functional images were acquired during resting state using accelerated gradient-echo echoplanar imaging (EPI) sequence: TR = 2000 ms, TE = 30 ms, FOV = 240 mm, 90° flip angle, 10 min acquisition, or 300 volumes, 1.9 × 1.9 × 2.0 mm voxel size and 56 slices with 10% slice gap. Participants lay still with their eyes open and fixated on a white crosshair presented on a black background of an LCD monitor viewed through a head coil-mounted mirror^24,112^. fMRI images were processed using FSL tools (FMRIB Software Library version 5.0; available at: https://fsl.fmrib.ox.ac.uk/fsl/fslwiki/)^113,114^. T1-weighted MPRAGE was extracted with brain extraction tool (BET)^115^ followed by the delineation of grey matter, white mater and cerebrospinal fluid voxels using a FAST segmentation^116^. Pre-processing of rs-fMRI data (using FSL FEAT tool^113,114^) included slice timing and motion correction, spatial smoothing (3 mm FWHM kernel), nuisance signal regression via GLM analysis, temporal band pass filtering (0.009–0.1 Hz), linear registration of functional images to structural images, and non-linear registration of structural images to MNI 152 template using 2 mm isotropic voxel resolution.

Subjects were excluded from analysis with absolute head displacement greater than 0.5 mm per TR (as estimated by FSL's MCFLIRT) for at least a third of the frames within a 10-min sequence. Remaining subjects displayed an absolute head displacement of on average 0.29 mm per scan (min: 0.06 mm, max: 2.49 mm). Affine registration across TRs was performed using MCFLIRT with default options. To account for partial volume effects and signal intensity changes due to linear motion and drift, an extended set of 12 motion parameters (six standard rotation and translation parameters at TR = 0 and TR = − 1, estimated with FEAT preprocessing) were included as explanatory variables (EV) for each TR in the nuisance regressor GLM analysis. Next, non-linear (> 12 dof) outliers in signal intensity due to head motion were identified using DVARS, with a boxplot cutoff threshold of 75th percentile plus IQR × 1.5^117^. For each outlier TR, an EV was added to the GLM analysis to partial out signal intensity in the TR. Lastly, mean white matter and cerebrospinal fluid signals averaged across all voxels were also included as the nuisance variables in the GLM analysis. Fully pre-processed rs-fMRI data were defined as residuals from the GLM models subsequently transformed to normalized MNI 152 space and resampled to 4 mm isotropic voxel resolution.

### Graph theory measures

Methods used to derive graph theory metrics followed exactly those previously described and any differences are clearly stated^118,119^. Weighted connectivity matrices for each brain network were computed across all subjects. Craddock’s 800 parcellated brain atlas^120^ was used to extract the mean time series signal from subjects’ BOLD fMRI signal at each parcel/region^121^; 662 regions common across subjects were identified. First a brain mask that included voxels with non-zero standard deviation values was generated for each subject. The masks were combined into a group mask, which represented voxels common to all subjects. The group mask was finally used on the Craddock’s 800 parcellated atlas, which resulted in 662 regions intersecting with group mask. For each subject a connectivity matrix consisting of 662 × 662 pairwise correlations between the average BOLD times series signals was computed and normalized using Fisher’s *Z*-transformation. Fisher Z-transformed correlations were then multiplied by their standard deviation (approximated as σ = 1/√(n − 3); n is number of samples comprising the BOLD signal), yielding standardized Z scores. Statistically significant positive correlations (*p* < 0.05) were identified and retained based on Bonferroni-corrected Z-thresholds for each subject’s FC matrix^122^. Negative and non-significant correlations where dropped^37^. Next, the thresholded Z scores were rescaled to represent connection weights ranging from 0 to 1. In contrast to binary graphs, where an arbitrary threshold is used to define a connection such that all connections with less than a specific correlation value (an absolute threshold) are set to 0 and all other connections are set to 1, in weighted graphs connection weights (i.e., rescaled statistically significant Z scores) is preserved, thus circumventing the need for thresholding^123^. Subject-wise weighted connectivity matrices were next derived for each of the 7 brain network maps, namely, visual, somatosensory, limbic, default mode, dorsal attention, ventral attention and frontoparietal (brain masks available at: https://surfer.nmr.mgh.harvard.edu/fsl-wiki/CorticalParcellationYeo2011)^37^. Adjacency matrices for each of the 7 brain networks are included as supplementary data D1.

Graph theoretic measures of global and local efficiency were derived for FPN, DAN, VAN and DMN based on their weighted connectivity matrices. Measure of global efficiency was calculated as the inverse of the average shortest paths of nodes belonging to a brain network^41,43^:

$${E}_{glob}^{w}= \frac{1}{n}\sum_{i\in N}\frac{{\sum }_{j\in N j\ne 1}{\left({d}_{ij}^{w}\right)}^{-1}}{n-1}$$

${E}_{glob}^{w}$ is the weighted global efficiency, N is the set of all the nodes in the brain network, ${d}_{ij}^{w}$ is the shortest weighted path length between nodes *i* and *j*^43^. Global efficiency provides a measure of how effectively information is integrated across the entire network^43^.

Local efficiency measures how interconnected neighboring nodes are to each other and was expressed as the inverse of the average shortest path connecting all neighbors of a given node^43^:

$${E}_{loc}^{w}= \frac{1}{2}\sum_{i\in N}{E}_{loc}^{w}\left(i\right)= \frac{1}{N}\sum_{i\in N}\frac{{\sum }_{j,h\in {G}_{i,}j \ne i}{w}_{ij}{w}_{ih}{\left[{d}_{jh}^{w}(Ni)\right]}^{-1}}{{k}_{i}^{w}({k}_{i}^{w}-1)},$$

where w_ij_ is the connection weight between *i* and *j*; d_jh_ (N_i_) is the shortest path length between *j* and *h* that is limited to neighbors of *i* and ${k}_{i}^{w}$ is the weighted degree, i.e., the number of links connected to node *i*^43^. Local efficiency was computed for each node within a brain network and divided by its total number of nodes. High local efficiency reflects the efficiency of information transfer within a module (here: a specific brain network).

### Adult-decision making competence battery (A-DMC)

To account for the multidimensional character of DMC^6,17^, we administered the most comprehensive battery of decision-making processes, which consisted of: (1) belief assessment that included consistency in risk perception (whether decision-making choices violate rules of probabilistic reasoning) and ability to recognize social norms (accurately judging normative beliefs of age-matched peers); (2) value assessment, comprising the ability to overcome sunk cost (considering future options not prior investments) and resist normatively irrelevant problem frames (resistance to framing), expressed as 5 minus mean absolute difference between related frames (e.g., gain and loss) of the same problem; (3) information integration, which measured the application of complex rules (applying decision rules); (4) metacognitive ability to identify the limitations of one’s knowledge (over/underconfidence)^6^. Scoring included: percentages of responses that adhered to the probability theory (on a 0–1 scale; consistency in risk perception); rank-order correlation (varying from − 1 to + 1) between the proportion of respondents judging several undesirable behaviors as “sometimes OK”, and decision-maker’s estimate of this proportion (recognizing social norms); the number of times when previous investments were correctly discounted (on a 1–6 point scale; resistance to sunk cost); the mean difference between scores on problems framed as a gain and a loss (0–5 point scale; resistance to framing); percent of problems solved by correctly applying rules (ranging from 0 to 1; applying decision rules); and the absolute difference between decision-maker’s confidence in their response accuracy to questions of general knowledge and the percentage of correct responses (higher scores represent better performance; over/underconfidence)^6^. Unweighted average of z-scores across six tasks were used as an index of overall DMC (A-DMC index)^6^. A-DMC battery has acceptable reliability and internal consistency^6^.

### Covariates

All analyses were adjusted for accelerometer wear time^124^. Remaining covariates were chosen based on their associations with FC and DMC, and included: age^125,126^, sex^127^, education (≤ High School or > High School)^6^, body mass index (BMI; kg m^−2^)^128^, IQ^6^, and aerobic fitness^5,37^. Height and weight were measured using a stadiometer (model 240; Seca, Hamburg, Germany) and a digital scale (WB-300 Plus; Tanita Tokyo, Japan). Fluid and crystallized intelligence were measured using Figure Series (Z scores representing the number of correct items)^129^ and Shipley-2 Vocabulary Scale (standard scores)^105^, respectively. Aerobic fitness was assessed with graded maximal exercise test on a treadmill^11^ using indirect computerized calorimetry (Parvo Medics TrueOne 2400) and a modified Balke protocol^130^. FFVO_2_peak was defined based on the test meeting ≥ 1 of the following criteria^11^: (1) a plateau in VO_2_ or failure to increase VO_2_ by 150 mL min^−1^ with increased workload^11^; (2) heart rate > 85% of predicted maximum^130^; (3) a rating of perceived exertion > 17^131^; or (4) a peak RER ≥ 1.10 (mL kg^−1^ min^−1^ relative to fat free mass)^132^. Fat free mass was measured using dual X-ray absorptiometry (DXA; Hologic QDR 4500A, software version 13.4.2, Bedford, MA, USA).

### Statistical analyses

**Figure 3:**
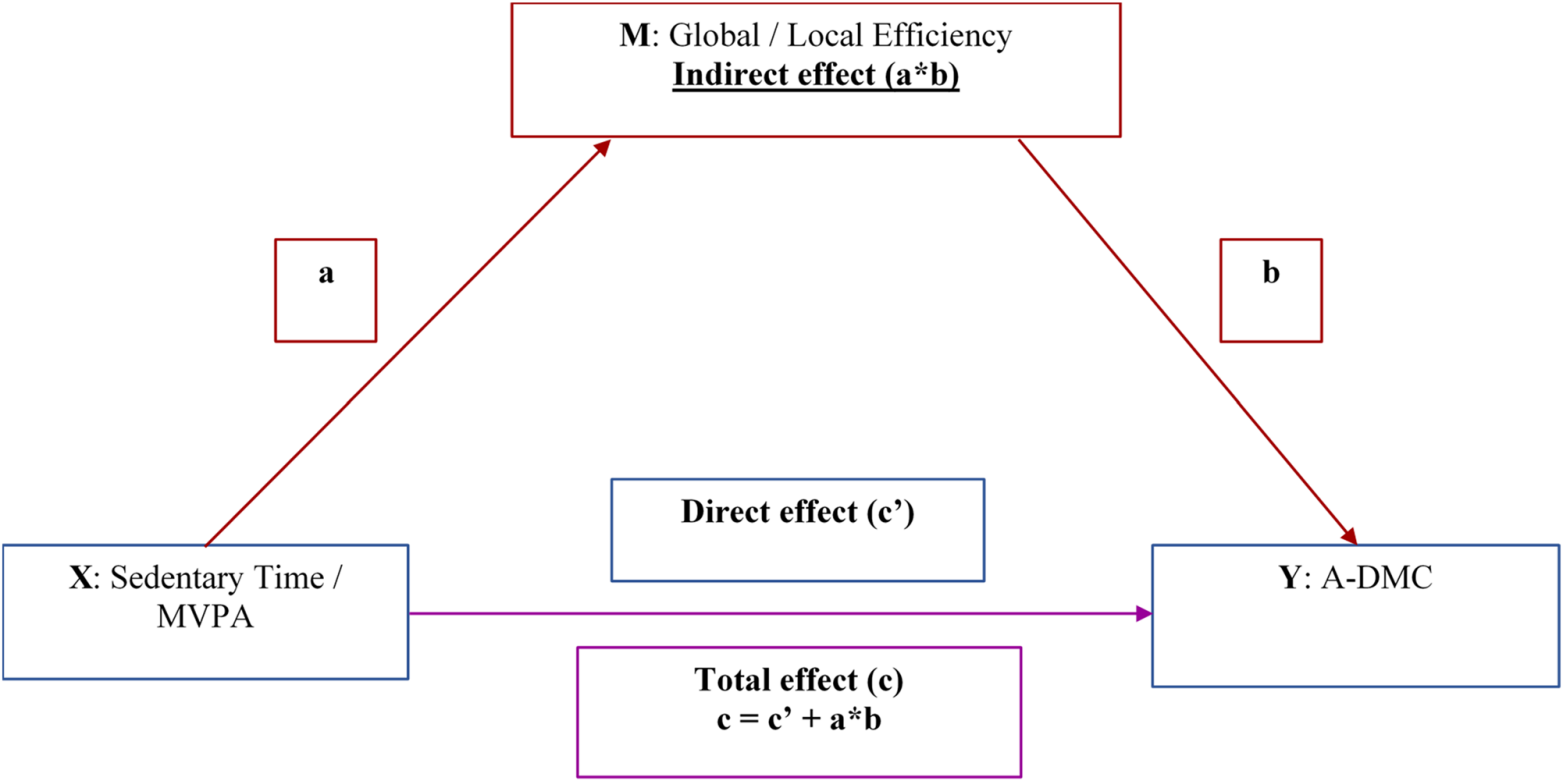
Mediated effects of global and local efficiency of intrinsic brain networks on the relationship between sedentary time and set shifting. Blue represents a direct effect of a predictor (X) on the outcome (Y), adjusting for a mediator (M); red represents an indirect effect of X on Y via M, which is a product of paths a and b; magenta represents the total effect of X on Y which is the sum of the indirect effect (red) and the direct effects (blue).

Global efficiency (FPN, DAN, VAN and DMN) and local efficiency (FPN) were positively skewed and transformed using Tukey power transformations. All A-DMC test scores (except for Z score) were negatively skewed, thus reflected and transformed using Tukey power transformations. All other variables were normally distributed. Associations between MVPA, sedentary time, FC in FPN, DAN, VAN, DMN, and A-DMC tests were inspected using multiple linear regression models. We first assessed the total effect of MVPA/sedentary time on A-DMC tests (path c), adjusting for age, sex, BMI, education, IQ and FFVO_2_peak (Model 1), and additionally adjusting for sedentary time or MVPA in Model 2. The total effect does not account for global or local efficiency of brain networks. Next, we tested mediating effects models. In step 1, global and local efficiency in each brain network were regressed on MVPA and sedentary time (path a, Fig. 3). Models 1 and 2 were conducted for each network. In step 2, performance on each of the A-DMC tests was regressed on global and local efficiency in brain networks which were significantly related to MVPA or sedentary time in step 1, using models 1 and 2 (path b, Fig. 3). In step 3, we formally tested the mediating effects of global and local efficiency of brain networks that were significantly related to MVPA or sedentary time and A-DMC tests. Mediating (indirect) effect was defined as a statistically significant interaction term between paths a and b (a*b)^58^. PROCESS macro (https://processmacro.org) was used to compute bootstrap confidence intervals for the indirect effect (10,000 samples)^58^. Direct effect was estimated by regressing A-DMC scores on MVPA/sedentary time, controlling for the covariates specified in Models 1 and 2, as well as global or local efficiency in a respective brain networks (path c′). Significance was set to α = 0.05. All *p* values reported in the text have been corrected for multiple comparisons using FDR^57^. Significance for indirect effects is defined as 95% bootstrap confidence intervals not containing a zero. Statistical analyses were conducted using IBM SPSS Statistical software (v. 25.0, IBM Corp., Armonk, NY, USA).

## Supplementary information

Supplementary file1
