# Supplementary material for: Opposing associations between sedentary time and decision-making competence in young adults revealed by functional connectivity in the dorsal attention network
Source: Sci Rep. 2020 Aug 19;10:13993. doi: 10.1038/s41598-020-70679-7 (PMC7438333; doi:10.1038/s41598-020-70679-7)
Supplement: Supplementary file 1 — Supplementary file1 [file 41598_2020_70679_MOESM1_ESM.docx]

Title: Opposing associations between sedentary time and decision-making competence in young adults revealed by functional connectivity in the dorsal attention network

Dominika M. Pindus,^1,2*^ Christopher E. Zwilling,^2^ Jennifer S. Jarrett,^4^ Tanveer Talukdar,^2^ Hillary Schwarb,^2,3^ Evan Anderson,^2,4^ Neal J. Cohen,^2,3,4^ Aron K. Barbey,^2,4^ Arthur F. Kramer^2,5^ and Charles H. Hillman^5,6^

^1^Dept. of Kinesiology and Community Health, University of Illinois at Urbana-Champaign, Urbana, IL;

^2^Beckman Institute for Advanced Science and Technology, University of Illinois at Urbana-Champaign, Urbana, IL

^3^Interdisciplinary Health Sciences Institute, University of Illinois at Urbana-Champaign, Urbana, IL

^4^Department of Psychology, University of Illinois at Urbana-Champaign, Urbana, IL

^5^Department of Psychology, Northeastern University, Boston, MA

^6^Department of Physical Therapy, Movement, & Rehabilitation Sciences, Northeastern University, Boston, MA

Corresponding author: Dominika M. Pindus

Department of Kinesiology and Community Health

University of Illinois at Urbana-Champaign

305 Louis Freer Hall

906 S Goodwin Avenue

Urbana, IL 61801

Email: pindus@illinois.edu

Submitted: 11/30/2019

Revised: 06/01/2020

Supplementary Table S1. Adult-Decision Making Competence tests regressed on sedentary time and daily moderate-to-vigorous physical activity uncorrected for false discovery rate

|  |  | **Consistency in Risk Perception** | **Recognizing Social Norms** | **Sunk Cost** | **Resistance to Framing** | **Applying Decision Rules** | **Over/**  **Under-confidence** | **ADMC-Z score** |
| --- | --- | --- | --- | --- | --- | --- | --- | --- |
| **Sedentary time** | ∆R^2^  β  (SE)  b | 0.01  -0.0003  0.0002  -0.10 | 0.00  0.0000  0.0002  0.02 | 0.00  0.0006  0.0008  0.05 | **0.03**  **-0.0004**  **0.0002**  **-0.16** | 0.01  -0.0004  0.0003  -0.09 | 0.00  0.0001  0.0002  0.03 | **0.02**  **0.0010**  **0.0005**  **0.13** |
| **Sedentary time adjusted for MVPA** | ∆R^2^  β  (SE)  b | 0.01  -0.0003  0.0002  -0.11 | 0.00  -0.0000  0.0002  -0.01 | 0.00  0.0007  0.0008  0.07 | **0.03**  **-0.0004**  **0.0002**  **-0.16** | 0.02  -0.0006  0.0003  -0.13 | 0.00  0.0000  0.0002  0.01 | **0.02**  **0.0013**  **0.0005**  **0.16** |
| **MVPA** | ∆R^2^  β  (SE)  b | 0.00  0.0000  0.0005  0.01 | 0.00  -0.0004  0.0005  -0.06 | 0.00  0.0007  0.0021  0.03 | 0.00  0.0001  0.0005  0.02 | 0.01  -0.0008  0.0007  -0.08 | 0.00  -0.0005  0.0006  -0.06 | 0.00  0.0012  0.0014  0.05 |
| **MVPA adjusted for sedentary time** | ∆R^2^  β  (SE)  b | 0.00  -0.0002  0.0005  -0.03 | 0.00  -0.0004  0.0005  -0.06 | 0.00  0.0013  0.0022  0.04 | 0.00  -0.0002  0.0005  -0.03 | 0.01  -0.0013  0.0008  -0.11 | 0.00  -0.0005  0.0007  -0.06 | 0.01  0.0024  0.0015  0.10 |

*Note.* All A-DMC test scores (except for ADMC Z-score) were negatively skewed, reflected and transformed using Tukey power transformations. Thus, negative values indicate a positive relationship. Abbreviations: ADMC: Adult-Decision Making Competence Battery [6]; MVPA: moderate-to-vigorous physical activity. Bolded values indicate significant associations (uncorrected). Resistance to framing regressed on sedentary time and sedentary time adjusted for MVPA: ANOVAs *Fs* ≥ 4.69, *p*s < 0.001; *t*s ≥ 2.40, *p*s ≤ 0.02. ADMC Z-score regressed on sedentary time and sedentary time adjusted for MVPA: ANOVAs *Fs* ≥ 8.25, *p*s < 0.001; *t*s ≥ 2.02, *p*s ≤ 0.045. All non-significant findings: *p*s *≥* 0.05 (uncorrected). Model: ADMC test = age + sex + education + BMI+ IQ + FFVO_2_peak + MVPA/Sedentary time

Supplementary Table S2. Global efficiency of intrinsic connectivity networks regressed on sedentary time and daily moderate-to-vigorous physical activity uncorrected for false discovery rate

|  |  | **FPN** | **DAN** | **VAN** | **DMN** |
| --- | --- | --- | --- | --- | --- |
| **Sedentary time** | ∆R^2^  β  (SE)  b | 0.0095  -0.0002  0.0001  -0.0999 | **0.0488**  **-0.0006**  **0.0002**  **-0.2266** | **0.0207**  **-0.0003**  **0.0002**  **-0.1476** | 0.0035  -0.0002  0.0002  -0.0605 |
| **Sedentary time adjusted for MVPA** | ∆R^2^  β  (SE)  b | 0.0107  -0.0002  0.0002  -0.1047 | **0.0375**  **-0.0005**  **0.0002**  **-0.1963** | **0.0287**  **-0.0004**  **0.0002**  **-0.1720** | 0.0073  -0.0002  0.0002  - 0.0864 |
| **MVPA** | ∆R^2^  β  (SE)  b | 0.0000  0.0000  0.0004  0.0015 | 0.0114  0.0008  0.0005  0.1108 | 0.0017  -0.0003  0.0005  -0.0424 | 0.0032  -0.0004  0.0005  -0.0587 |
| **MVPA adjusted for sedentary time** | ∆R^2^  β  (SE)  b | 0.0014  -0.0002  0.0004  -0.0384 | 0.0007  0.0002  0.0005  0.0260 | 0.0103  -0.0007  0.0005  -0.1038 | 0.0071  -0.0006  0.0005  -0.0861 |

*Note.* Global efficiency in intrinsic functional brain networks was transformed using Tukey power transformations. All physical behavior variables were adjusted for accelerometer wear time. Abbreviations: MVPA: moderate-to-vigorous physical activity, FPN: frontoparietal network, DAN: dorsal attention network, VAN: ventral attention network, DMN: default mode network. Bolded values indicate significant associations (uncorrected). Global efficiency in the DAN regressed on sedentary time and sedentary time adjusted for MVPA: ANOVAs *Fs* ≥ 2.32, *p*s ≤ 0.021; *t*s ≥ 2.81, *p*s ≤ 0.005. All non-significant findings: *p*s *≥* 0.13 (uncorrected). Model: Global efficiency of a brain network = age + sex + education + BMI+ IQ + FFVO_2_peak + MVPA/Sedentary time

Supplementary Table S3. Local efficiency of intrinsic connectivity networks regressed on sedentary time and daily moderate-to-vigorous physical activity uncorrected for false discovery rate

|  |  | **FPN** | **DAN** | **VAN** | **DMN** |
| --- | --- | --- | --- | --- | --- |
| **Sedentary time** | ∆R^2^  β  (SE)  B | 0.0002  -0.0000  0.0001  -0.0127 | **0.0329**  **-0.0001**  **0.0000**  **-0.1861** | 0.0004  -0.0000  0.0000  -0.0194 | 0.0015  0.0000  0.0000  0.0392 |
| **Sedentary time adjusted for MVPA** | ∆R^2^  β  (SE)  B | 0.0003  -0.0000  0.0002  -0.0166 | 0.0158  -0.0001  0.0000  -0.1276 | 0.0029  -0.0000  0.0000  -0.0546 | 0.0005  0.0000  0.0000  0.0226 |
| **MVPA** | ∆R^2^  β  (SE)  B | 0.0001  -0.0000  0.0004  -0.0084 | 0.0296†  0.0003  0.0001  0.1783 | 0.0074  -0.0001  0.0001  -0.0890 | 0.0022  -0.0001  0.0001  -0.0489 |
| **MVPA**  **adjusted for sedentary time** | ∆R^2^  β  (SE)  B | 0.0002  -0.0001  0.0004  -0.0140 | 0.0123  0.0002  0.0001  0.1134 | 0.0098  -0.0002  0.0001  -0.1016 | 0.0012  -0.0000  0.0001  -0.0358 |

*Note.* Local efficiency in frontoparietal networks was transformed using Tukey power transformations. Data for all other intrinsic connectivity brain networks was untransformed. All physical behavior variables were adjusted for accelerometer wear time. Abbreviations: MVPA: moderate-to-vigorous physical activity, FPN: frontoparietal, DAN: dorsal attention network, VAN: ventral attention network, DMN: default mode network. Bolded values indicate significant associations (uncorrected). Local efficiency in the DAN regressed on sedentary time: ANOVA *F*(8, 192) = 2.08, *p* = 0.04; *t*(192) = 2.62, *p* = 0.009. Local efficiency in the DAN regressed on MVPA: *F*(8, 192) = 1.99, *p* = 0.050; *t*(192) = 2.48, *p* = 0.014. All non-significant findings: *p*s *≥* 0.07 (uncorrected). Model: Local efficiency of a brain network = age + sex + education + BMI+ IQ + FFVO_2_peak + MVPA/Sedentary time.

Supplementary Data D1. Adjacency matrices for seven intrinsic connectivity brain networks: visual, somatosensory, limbic, default mode, dorsal attention, ventral attention and frontoparietal.
